# A novel superficial temporal artery patency concept of cerebral revascularization for patients with moyamoya disease: a multicenter study

**DOI:** 10.1186/s41016-025-00424-4

**Published:** 2026-02-26

**Authors:** Zhiyong Shi, Xin Li, Xinhua Chen, Lingyun Wu, Rong Wang, Chunhua Hang, Yongbo Yang, Dong Zhang

**Affiliations:** 1 Department of Neurosurgery, Nanjing Drum Tower Hospital, Nanjing University Medical school, Nanjing, Jiangsu Province 210008 People’s Republic of China; 2 Department of Neurosurgery, Beijing Tiantan Hospital, Capital Medical University, Beijing, MN 100070 People’s Republic of China; 3 Department of Neurosurgery, Dongcheng District, Beijing Hospital, No 1 Dahua Road, Beijing, MN 100730 China

**Keywords:** Moyamoya disease (MMD), Cerebral revascularization, Surgical modified technique, Clinical prognosis

## Abstract

**Background:**

To summarize the clinical and radiological outcomes of a novel cerebral revascularization technique based on the superficial temporal artery patency concept (STAPC) in patients with moyamoya disease (MMD).

**Methods:**

A retrospective review was conducted of adult patients with MMD treated at Beijing Hospital and Nanjing Drum Tower Hospital between January 2019 and December 2021. The cohort comprised 170 patients who underwent superficial temporal artery–middle cerebral artery bypass with encephalo-duro-arterio-synangiosis (EDAS) (STA-MCA/EDAS), and 133 who underwent EDAS alone. Radiological follow-up included computed tomography (CT) angiography (CTA) to assess bypass patency and CT perfusion (CTP) for hemodynamic staging at 3 and 12 months post-revascularization. Clinical follow-up recorded perioperative complications and recurrent stroke events that occurred > 12 months postoperativerly.

**Results:**

Of the 303 patients, 37 cases (12.21%) had perioperative complications including 27 cases (15.9%) in the STA-MCA/EDAS group and 10 cases (7.5%) in the EDAS group. Perfusion improvement was observed in 23.76% of patients (25.7% in the STA-MCA/EDAS group and 17.8% in the EDAS group) at 3 months postsurgical, and in 40.17% of patients (48.68% in the STA-MCA/EDAS group and 24.39% in the EDAS group) at 12 months postsurgical. Bypass patency was observed in 95.29% of patients (96.1% in the STA-MCA/EDAS group and 93.5% in the EDAS group) at 3 months postoperative, and in 96.43% patients (95.2% in the STA-MCA/EDAS group and 97.1% in the EDAS group) at 12 months postoperative. Of the 249 patients with a median follow-up period of 50 months (range 12–70 months), 40 cases (16.06%, 3.85% per year) had recurrent stroke events including 22 (15.41%, 3.69% per year) in the STA-MCA/EDAS group and 18 (16.67%, 3.91% per year) in the EDAS group.

**Conclusions:**

Cerebral revascularization using STAPC is an acceptable surgical strategy for preventing stroke recurrence in patients with MMD.

**Supplementary Information:**

The online version contains supplementary material available at 10.1186/s41016-025-00424-4.

## Background

Moyamoya disease (MMD) is a rare cerebrovascular disease in Asia, characterized by chronic progressive stenosis of the intracranial arteries and the formation of moyamoya vessels at the base of the brain [1]. The natural course of MMD is poor, which could result in brain ischemia, hemorrhage, and cognitive impairment [2–4]. Extracranial-intracranial (EC-IC) bypass, characterized by superficial temporal artery-middle cerebral artery (STA-MCA) bypass, encephalo-duro-arterio-synangiosis (EDAS) bypass, was considered optimal treatment for patients with MMD, contributing to reduce stroke recurrence and preserve cognitive function during clinical follow-up [2, 5–7]. In surgical for practice, we proposed a new STA patency concept (STAPC) for cerebral revascularization, focusing on STA patency and targeting recipient artery (t-RA), to achieve the patency of STA and overlap of neovascularization for ischemic brain tissue. In this study, we aimed to illustrate the surgical details of STAPC and present a series of radiological and clinical follow-up results.

## Methods

### Patient selection

This study was approved by the Ethics Committees of Beijing Hospital and Nanjing Drum Tower Hospital. Informed consent was obtained from all participants. The inclusion criteria were as follows: (1) met the diagnosis criteria of MMD proposed by the Research Committee on Spontaneous Occlusion of the Circle of Willis of the Ministry of Health, Labor, and Welfare, Japan [8]; (2) treated with by EC-IC bypass based on STAPC; (3) operated hemisphere evaluated by intraoperative microvascular doppler ultrasonography (MDU) and FLOW 800 indocyanine green (ICG) examinations; (4) patient underwent CT perfusion (CTP), and CT angiography (CTA) or digital subtraction angiography (DSA) examination during follow-up; and (5) patient aged over 18 years. Those patients with age less than 18 years old with moyamoya syndrome (MMS), posterior circulation ischemia, incomplete data, and mental disorders were excluded.

A total of 303 adults MMD patients were recruited between January 2019 to December 2021, including 177 cases in the STA-MCA/EDAS group and 133 cases in the EDAS group. The demographics of general information, radiological images, incidence of newly developed complications, and clinical follow-up outcomes were collected.

### Data collection and analysis

#### General information

Patient information included sex, age, symptom onset, hypertension, diabetes, smoking history, modified Rankin Scale (mRS) score, intracranial aneurysm, Suzuki stage, and hemispheric pre-infarction stage, etc.

#### DSA images

According to a previous report, the Suzuki stage was divided into three grades: (1) early stage, hemisphere with Suzuki I and II; (2) mild stage, hemisphere with Suzuki III and IV; (3) advanced stage, hemisphere corresponding to Suzuki V and VI [9]. According to Matsushima grades, neovascularization exceeding 2/3, 1/3 ~ 2/3, and < 1/3 MCA territory were defined as grade A, B, and C respectively [9]. Bypass patency rate were also recorded.

#### CTP images

The stage of hemispheric perfusion was evaluated based on CTP [10], using the cerebellar cortex as a reference. CTP parameters included regional cerebral blood volume (rCBV), regional cerebral blood flow (rCBF), mean transit time (MTT), and time to peak (TTP). The criteria of pre-infarction period were as follows: Stage Ia was delayed TTP, normal MTT, rCBF, and rCBV. Stage Ib was delayed TTP and MTT, whereas rCBF and rCBV were normal or slightly increased. Stage IIa was delayed TTP and MTT, decreased rCBF, but normal or slightly decreased rCBV. Stage IIb was delayed TTP and MTT and decreased rCBF and rCBV.

#### Strategy of STAPC

The STAPC prioritizes preservation of the functional STA as the primary donor artery, strategically directing its blood supply (via direct/indirect means) to establish durable EC-IC collateral neovascularization. The fundamental strategy of STAPC is to preserve STA patency, evaluate the ratio of STA and recipient artery (RA) diameter, and establish durable collateral pathways.

#### Surgical techniques of STAPC

*Step 1*. The patient was in a supine position, keeping the zygomatic bone at the highest point. Doppler probe was used to mark the frontal and parietal branches of STA from the root of the zygomatic arch. *Step 2.* The fascia of STA and its branches (tailored STA) and used papaverine to prevent vasospasm. *Step 3*. Made a “T” shape incision of the temporal muscle, opened the skull with the sylvian fissure as the center, and exposed the frontal, parietal, and temporal lobes, during which it was important to avoid damage to the middle meningeal artery (MMA). Cut the dura mater in the shape of “+” and minimized the amount of electrocoagulation to stop bleeding. *Step 4*. Intraoperative MDU was employed to monitor flow velocity, and the artery exhibiting the minimal cortical flow velocity or the slowest fluorescence angiographic filling was selected as the target RA. Besides, the diameter ratio of the STA and RA must be assessed. For cases with an STA/RA ratio > 2 or RA diameter < 1 mm, EDAS was preferred. For cases with a ratio < 2 or RA diameter > 1 mm, STA-MCA/EDAS was prioritized. *Step 5.* The STA was cut with a “fish mouth cut”, with the length being about 2.5–4 mm. The RA was cut with the help of two temporary aneurysm clips. Used a 10–0 nylon thread to suture the heel and tip portion first and completed the anastomosis with the fashion of interrupted tip or continuous suture. After the anastomosis was completed and the temporary clip was removed, indocyanine green fluoroscopy (ICG) or Doppler ultrasound was used to detect the patency of the bypass. If the bypass was with poor patency, it was necessary to ascertain the cause and deal with it accordingly. *Step 6*. Sutured the STA branch with the incised arachnoid membrane with a 10–0 nylon thread. The outer layer of the dura flap was turned and folded inward to overlap the surface of the brain and sutured it with the free edge of the dura matter.

**Fig. 1:**
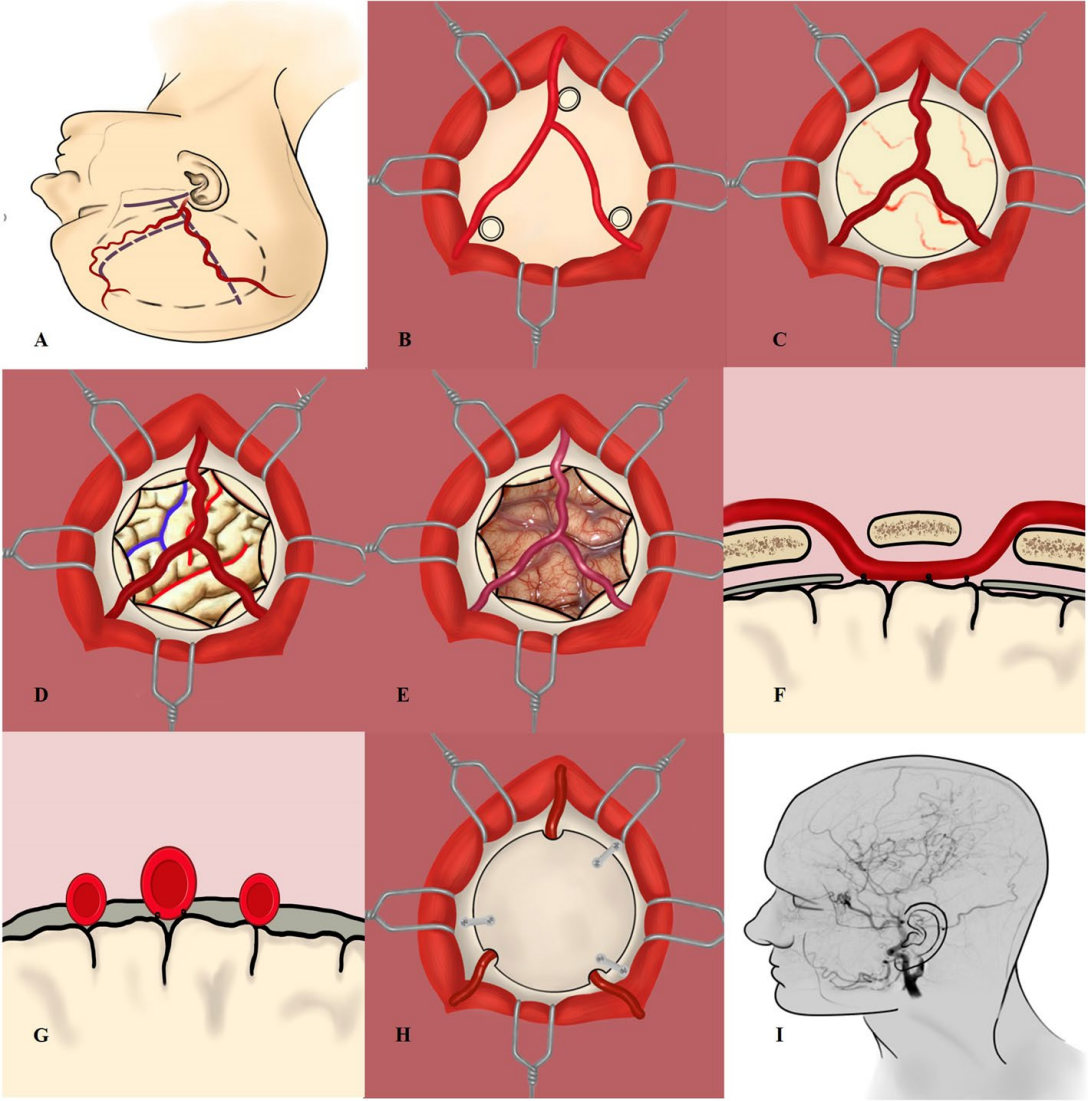
The illustration of EDAS with two branches of STA. **A** Make an incision of Y shape along the STA and the circular (dotted line) was the scope of operation. **B** The Y-shaped STA and adjacent bone holes were seen during the operation. **C** After bone flap removal, the STA and MMA were visible (dura unopened). **D**, **E** The illustration and surgical view of a 30-year-old male patient with MMD undergoing EDAS. The red line represented the sylvian fissure and central sulcus. The blue line indicated the central sulcus and inferior sulcus of the frontal lobe. **F**, **G** Used a 10–0 nylon thread to suture the STA to dura mater and STA was under the bone flap (cross-section view). **H** Put the bone flap back and STA branches passed under it through the bone hole. **I** Postoperative angiography showed that the intracranial arteries were well compensated

**Fig. 2:**
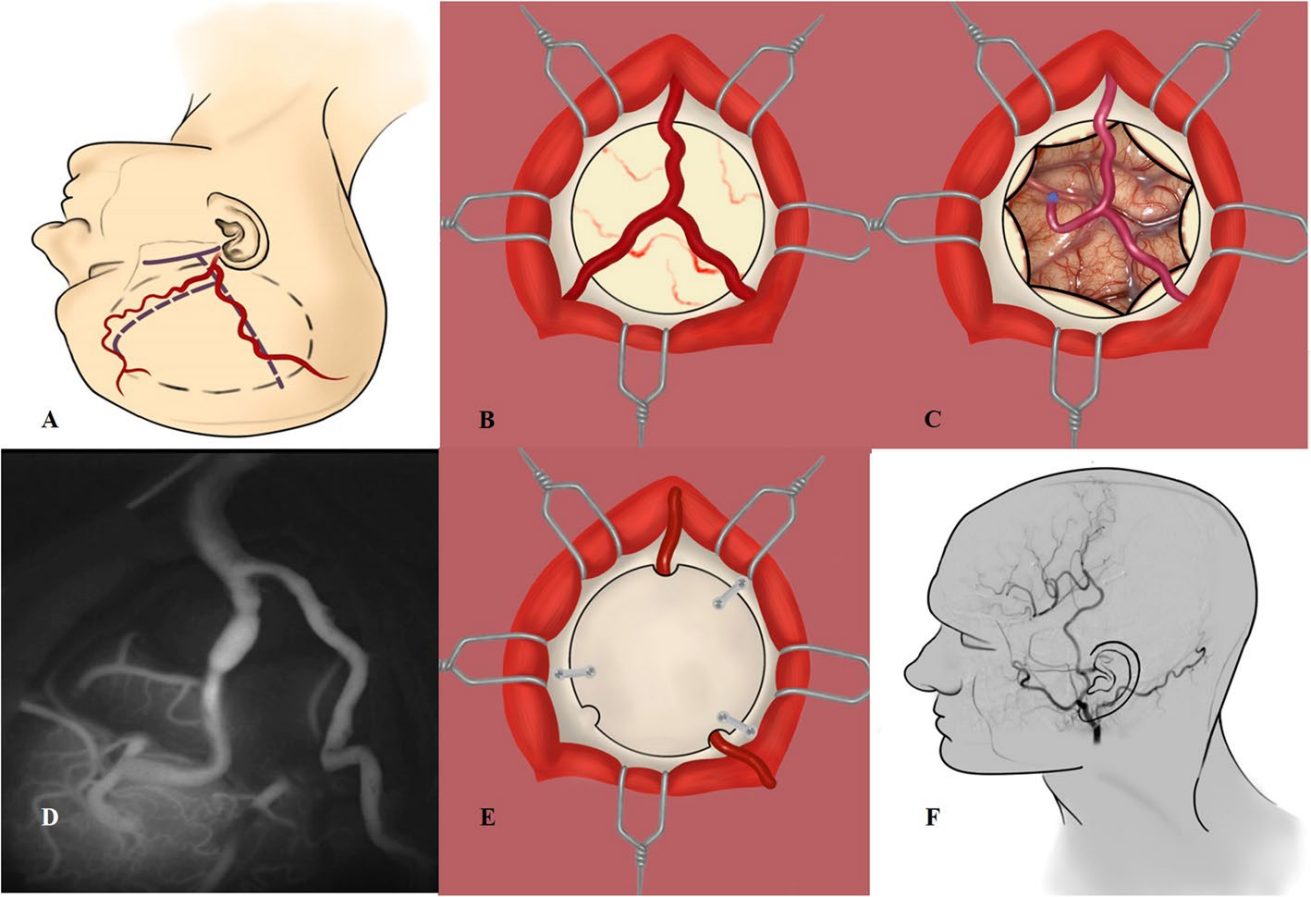
The illustration of STA-MCA/EDAS. **A** Make an incision of Y shape along the STA, and the circular (dotted line) was the scope of operation. **B** After bone flap removal, the STA and MMA were visible (dura unopened). **C** Used a 10–0 nylon thread to suture the anastomosis between STA donor artery and recipient artery (M4) with the fashion of interrupted tip. **D** After the anastomosis was completed and the temporary clip was removed, intraoperative indocyanine green fluoroscopy was used to detect the patency of the bypass. **E** Put the bone flap back, and the posterior branch of STA passed under it through the bone hole. **F** Postoperative angiography showed that the bypass was patent and the intracranial vessels were well developed

EDAS included surgical steps from 1 to 4 and 6 (Fig. 1), whereas STA-MCA/EDAS steps from 1 to 6 (Fig. 2). Surgical techniques and tips were shown in *supplementary materials.*

#### Postoperative complications

**Fig. 3:**
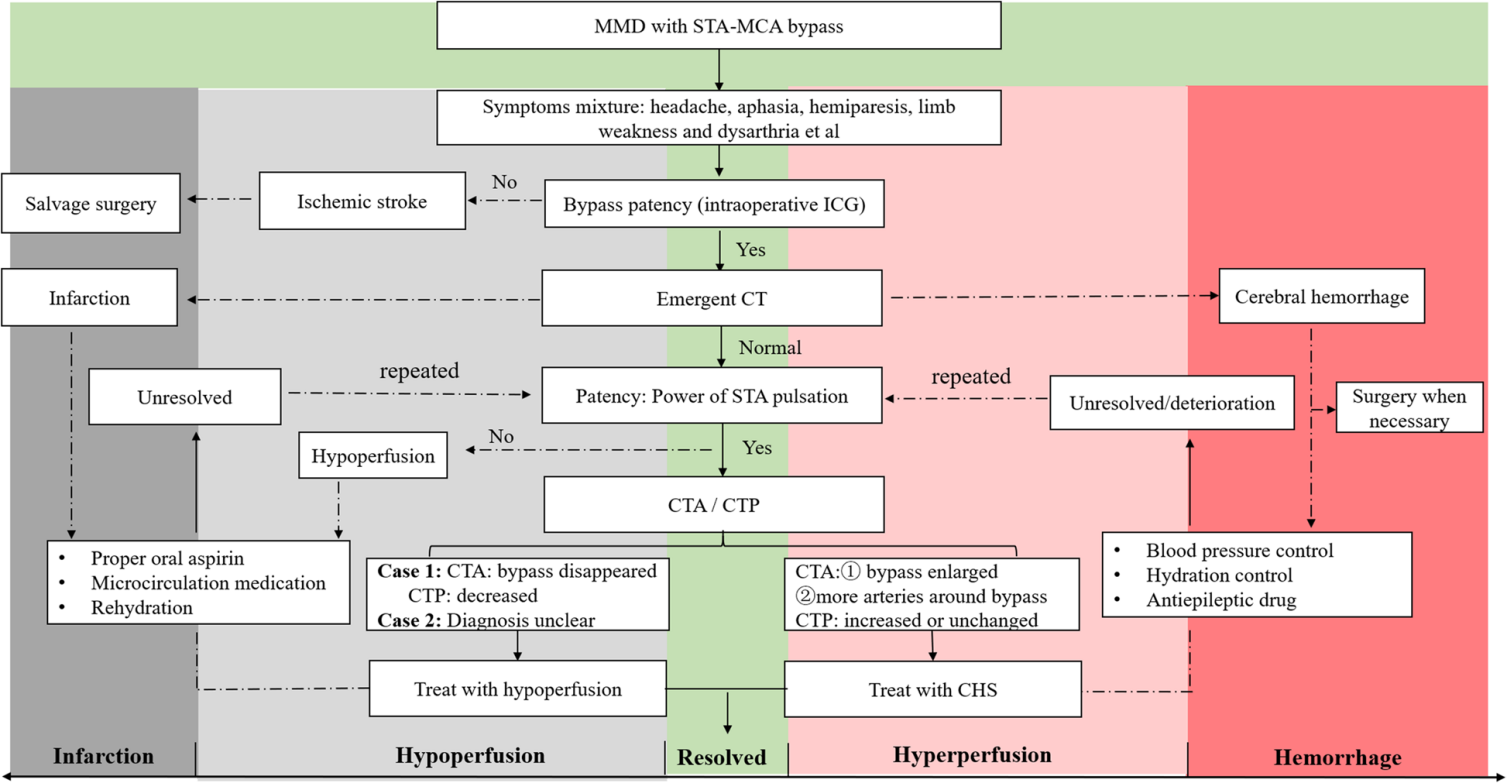
The diagram of treatment of postoperative neurological complications in our center

The new occurrence of postoperative complications was carefully recorded, including newly developed cerebral hemorrhage or infarction, cerebral hyperperfusion syndrome (CHS), subdural effusion, wound infection, and seizure. For cases exhibiting new discomfort after surgery, CTA/CTP examinations were performed to evaluate the bypass patency and cerebral perfusion when necessary. The diagram of treatment of postoperative neurological complications was summarized in Fig. 3.

### Clinical follow-up protocol

#### Radiological follow-up

Bypass patency was evaluated using CTA at 3 months postoperatively and DSA at 12 months postoperatively. Relative to preoperative hemispheric CTP staging, patients were classified into three categories based on postoperative changes: “improvement” (decreased perfusion stage), “no change” (unchanged stage), or “deterioration” (increased stage).

#### Clinical follow-up

Clinical follow-up of each patient was conducted by telephone or clinic interviews more than 12 months after surgery. Recurrent cerebral hemorrhage or ischemia, onset of epilepsy, and death were recorded during follow-up. The mRS score was performed to evaluate neurological status at discharge and at clinical follow-up. The patients with mRS score > 3, 2 ~ 3, and < 2 were defined as severe disability, moderate disability, and normal function, respectively [11].

### Statistical analysis

This observational study adopted a retrospective cohort design, utilizing electronic medical records from Beijing Hospital and Nanjing Drum Tower Hospital between January 2019 and December 2021. The primary outcomes included the incidence of postoperative complication before discharge, cerebral perfusion at 3 and 12 months postoperatively, and long-term clinical follow-up outcomes (minimal 12 months). Data were presented as frequencies (percentages).

## Results

### General patient characteristic

We identified 303 MMD cases utilizing STAPC, with an average age of 43 ± 10 years (range 16 ~ 75 years). The initial onset symptoms at admission were cerebral ischemia, brain hemorrhage, headache, others, and epilepsy, in 171, 105, 2, 14, and 11 cases, respectively. Of the 244 patients with cerebral angiography, 69.97% patients presented with Suzuki Stage 3, 4, and 5. Patient characteristics were detailed in Table 1.

**Table 1:** A summary of patient characteristics

Characteristic	No of patients (%)
Total	303
Gender (female/male)	157/146
Mean age (years)	43.32 ± 10.09
Clinical type	
Ischemic symptom	171 (56.43%)
Infarction	125
TIA	46
Type of hemorrhage	105 (34.65%)
SAH	10
ICH	32
IVH	40
SAH + ICH	2
SAH + IVH	1
ICH + IVH	17
Uncertain	3
Epilepsy	2 (0.66%)
Headache	14 (4.62%)
Others	11 (3.63%)
mRS scores on admission	
0	26
1	221
2	37
3	13
4	3
5	3
Hypertension	125 (41.25%)
Diabetes	33 (10.9%)
Smoking	59 (19.47%)
Intracranial aneurysm	9 (2.97%)
Central type	6
Peripheral type	3
Suzuki stage	244
1	3
2	15
3	80
4	75
5	57
6	14
Pre-infarction stage	303 (100%)
Ia	15
Ib	99
IIa	116
IIb	73
Surgical revascularization	
Combined bypass	170 (56.1%)
EDAS	133 (43.9%)

*TIA* transient ischemic attack, *SAH* subarachnoid hemorrhage, *IVH* intraventricular hemorrhage, *ICH* intracerebral hemorrhage, *mRS* modified Rankin Scale, *EDAS* encephalo-duro-arterio-synangiosis

### Postoperative outcome

Of the 303 patients recruited, 37 cases (12.21%) had postoperative complication. Of the 170 patients in the STA-MCA/EDAS group, 27 (15.9%) cases presented with perioperative complications, including cerebral infarctions in 15 cases (8.8%), cerebral hemorrhage in 5 cases (2.9%), CHS in 20 cases (11.7%), subdural effusion in 3 cases (1.8%), and skin infection in 2 cases (1.2%). No patient presented with epilepsy or hydrocephalus. Of the 133 patients in the EDAS group, 10 patients (7.5%) manifested with perioperative complications, including 6 cerebral infarctions (4.5%), 4 subdural effusions (3.0%), and 8 skin infections (6.0%). There was no patient presenting with new bleeding, CHS, epilepsy, or hydrocephalus (Table 2).

**Table 2:** Outcome of perioperative complications and mRS scores

Variables	STA-MCA/EDAS (*n* = 170)	EDAS (*n* = 133)	Total (*n* = 303)
Postoperative complication (*n*, %)	27 (15.9%)	10 (7.5%)	37 (12.21%)
New cerebral infarction (*n*, %)	15 (8.8%)	6 (4.5%)	21 (6.93%)
New hemorrhage (*n*, %)	5 (2.9%)	0	5 (1.65%)
CHS (*n*, %)	20 (11.8%)	0	20 (6.6%)
Subdural effusion (*n*, %)	3 (1.8%)	4 (3.0%)	7 (2.31%)
Skin infection (*n*, %)	2 (1.2%)	8 (6.0%)	10 (3.3%)
Epilepsy (*n*, %)	0	0	0
Hydrocephalus (*n*, %)	0	0	0
mRS scores at discharge			
Normal (*n*, %)	135 (79.4%)	96 (72.2%)	231 (76.23%)
Moderate disability (*n*, %)	31 (18.23%)	31 (23.3%)	62 (20.46%)
Severe disability (*n*, %)	4 (2.35%)	6 (4.5%)	10 (3.3%)
Clinical outcome at discharge (*n*, %)			
Deterioration	12 (7.07%)	3 (2.25%)	15 (4.95%)
No change	132 (77.64%)	95 (71.43%)	227 (74.92%)
Improvement	26 (15.29%)	35 (26.32%)	61 (20.13%)

*mRS* modified Rankin Scale, *CHS* cerebral hyperperfusion syndrome

According to mRS scores at discharge, patients with severe disability, slight to moderate disability, and no disability were 4 patients (2.35%), 31 patients (18.23%), and 135 patients (79.4%) in the STA-MCA/EDAS group, and 6 patients (4.5%), 31 patients (23.3%), and 96 patients (72.2%) in the EDAS group, respectively. According to the comparison of mRS scores, the patients with deterioration, unchanged, and improvement were 15 (4.95%), 227 (74.92%), and 61 (20.13%), respectively (Table 2).

#### Radiological follow-up

Perfusion improvement was achieved in 23.76% of patients (25.7% in the STA-MCA/EDAS group and 17.8% in the EDAS group) at postsurgical 3 months, and 40.17% patients (48.68% in the STA-MCA/EDAS group and 24.39% in the EDAS group) at postsurgical 12 months. Patients with unchanged perfusion were 69.06% and 54.7% at postsurgical 3 and 12 months (Table 3). Bypass patency was achieved in 95.29% patients (96.1% in the STA-MCA/EDAS group and 93.5% in the EDAS group) at postsurgical 3 months, and 96.43% patients (95.2% in the STA-MCA/EDAS group and 97.1% in the EDAS group) at postsurgical 12 months (Table 4).

**Table 3:** Comparison of cerebral perfusion change based on CTP pre-infarction stage for MMD patients (*n*, %)

Variables	Postsurgical 3 months			Postsurgical 12 months		
**Perfusion outcome**	**Total (*n* = 181)**	**STA-MCA/EDAS (*n* = 136)**	**EDAS (*n* = 45)**	**STA-MCA/EDAS (*n* = 76)**	**EDAS (*n* = 41)**	**Total (*n* = 117)**
Improved	43 (23.76%)	35 (25.7%)	8 (17.8%)	37 (48.68%)	10 (24.39%)	47 (40.17%)
Unchanged	125 (69.06%)	92 (67.6%)	33 (73.3%)	35 (46.1%)	29 (70.73%)	64 (54.7%)
Deteriorated	13 (7.18%)	9 (6.7%)	4 (8.9%)	4 (5.22%)	2 (4.88%)	6 (5.13%)

**Table 4:** Comparison of bypass patency for patients with MMD (*n*, %)

Variables	Postsurgical 3 months			Postsurgical 12 months		
**Bypass outcome**	**Total (*n* = 191)**	**STA-MCA/EDAS (*n* = 129)**	**EDAS (*n* = 62)**	**STA-MCA/EDAS (*n* = 21)**	**EDAS (*n* = 35)**	**Total (*n* = 56)**
Occlusion	9 (4.71%)	5 (3.9%)	4 (6.5%)	1 (4.8%)	2 (5.71%)	3 (5.35%)
Patency	182 (95.29%)	124 (96.1%)	58 (93.5%)	20 (95.2%)	33 (94.29%)	53 (94.65%)

#### Clinical follow-up

Of the 249 patients with the median follow-up period of 50 months (range 12–70 months), 40 cases (16.06%, 3.85 per year) had new neurological complications, including 22 (15.41%) in the STA-MCA/EDAS group and 18 (16.67%) in the EDAS group. Of 141 patients in the STA-MCA/EDAS group, 22 patients (15.41%, 3.69% per year) presented with recurrent stroke events, including ischemic events (TIAs or cerebral infarctions) in 16 patients (11.3%), intracranial hemorrhage in 6 patients (4.3%), and rebleeding-related death in 3 patients (2.1%). Of the 108 cases in the EDAS group, recurrent stroke events occurred in 18 cases (16.67%, 3.92% per year), including 15 cases (12.7%) with ischemic events and 3 cases (2.8%) with rehemorrhage. The annual risk of newly developed stroke events for adult MMD patients was 3.86% in the STA-MCA/EDAS group and 3.92% in the EDAS group, respectively. According to mRS scores at follow-up, patients with severe disability, slight to moderate disability, and no disability were 3 patients (2.13%), 10 patients (7.09%), and 135 patients (88.65%) in the STA-MCA/EDAS group and 8 patients (7.41%), 14 patients (12.96%), and 84 patients (77.78%) in the EDAS group, respectively (Table 5).

**Table 5:** Long-term outcomes of 249 patients with 1-year minimum follow-up

Variables	STA-MCA/EDAS (*n* = 141)	EDAS (*n* = 108)	Total (*n* = 249)
Duration of follow-up (months)	50 (13, 70)	51 (12,67)	50 (12,70)
Follow-up events (*n*, %)	22 (15.41%)	18 (16.67%)	40 (16.06%)
Ischemic events	16 (11.3%)	15 (12.7%)	31 (12.45%)
Hemorrhage	6 (4.3%)	3 (2.8%)	9 (3.61%)
Death due to bleeding	3 (2.1%)	2 (1.9%)	5 (2.01%)
Stroke recurrence (%, per yrs)	3.69%	3.92%	3.85%
mRS scores at follow-up (*n*, %)			
Normal (*n*, %)	125 (88.65%)	84 (77.78%)	209 (83.94%)
Moderate disability (*n*, %)	10 (7.09%)	14 (12.96%)	24 (9.64%)
Severe disability (*n*, %)	3 (2.13%)	8 (7.41%)	11 (4.42%)
Death (*n*, %)	3 (2.13%)	2 (1.85%)	5 (2.0%)

*mRS* modified Rankin Scale

## Discussion

Previous literature reported that EC-IC bypass was the main treatment for MMD, which could effectively prevent ischemic stroke and improve prognosis, reducing the 5-year rebleeding rate from 31.6% to 11.9% [12]. At present, a variety of surgical treatments for MMD were available, with STA-MCA bypass or EDAS as the mainstream [13]. In our center, we mainly carried out cerebral revascularization with STA management as the core, and STA-MCA/EDAS and EDAS surgery were produced based on an individualized cerebral revascularization strategy. This study systematically explored the application of the STAPC in cerebral revascularization for MMD, analyzing multiple dimensions including technical details, postoperative complications, imaging characteristics, and follow-up of clinical outcomes. Compared with the classic EC-IC bypass, this work particularly emphasized maximizing the perfusion potential of the STA in MMD. To our knowledge, no systematic reports of a similar technical concept existed in the literature.

According to previously published research, the incidence of postoperative ischemic stroke after cerebral revascularization for MMD patients was about 7.4% ~ 22% [14–16]. In our study, 6.93% patients had new cerebral infarction, which was consistent with previous reports. Due to the long operation time, temporary occlusion of blood flow and abrupt hemodynamic changes, the incidence of neurological dysfunction for direct bypass was higher than that of indirect ones [17–19]. Preoperative acute cerebral infarction and poor cerebral blood supply were independent risk factors for the occurrence of ischemic stroke [20]. The author advises against overly aggressive blood pressure management within the normal range following bypass completion, especially in elderly patients, those with poor vascular conditions, severe hypertension, or incomplete recovery from general anesthesia. We hypothesized that excessive intraoperative or early postoperative blood pressure control may compromise cerebrovascular autoregulation and contribute to postoperative infarction in distant vascular territories, such as the contralateral hemisphere or ipsilateral anterior cerebral artery/posterior cerebral artery territories, especially in patients at high risk of cerebral ischemia. This hypothesis has not been previously reported in the literature and requires further investigation for validation in future studies.

Based on previous literature, the incidence of CHS was about 21.5% ~ 50.0%, and most of them were reversible [21, 22]. CHS was mostly caused by the contradiction between the impaired regulation of intracranial arteries, increased vascular permeability, and the sudden increase of blood flow through the bypass artery [23, 24]. In our research, 20 patients (11.8%) in the STA-MCA/EDAS group had postoperative CHS, of which 2.9% patients developed new hemorrhage. Recent studies have confirmed that modifications in surgical techniques, such as side-to-side anastomosis and one donor two recipients (1D2R) bypass, can reduce the incidence of postoperative CHS [25, 26]. Moreover, the appropriate use of antiplatelet drugs with aspirin, free radical scavengers with edaravone, and MMP-9 inhibitors with minocycline could also reduce the occurrence of CHS [27, 28]. Besides, the authors speculated that maintaining stable postoperative blood pressure and mitigating adverse personal behaviors, such as reducing mobile phone use, ensuring a quiet resting environment, limiting family visits, and controlling excessive speech activity, may also contribute significantly to preventing postoperative CHS. To the best of our knowledge, this aspect has not been previously reported, and further clinical studies are warranted to validate these observations.

According to previous reports, postoperative occlusion of donor vessels, especially STA after revascularization, was another potential complication, with the risk factors of the thrombus within anastomosis, imbalanced pressure gradient between donor and recipient vessels, and the unintentional entrapment of the graft vessels [29]. Amin-Hanjani et al. reported that if the competitive relationship between blood flow of the bypass artery and the collateral compensation was created, it would lead to atrophy or even occlusion of the bypass artery [18]. If the indirect bypass artery was occluded, a second salvage revascularization was required [30]. In our research, about 95% patients had patent bypass at postsurgical 3 and 12 months. When performing cerebral revascularization in our center, emphasis was placed on the patency of STA and simultaneous revascularization of direct or indirect bypass. During the procedure, a thick heparin needle and continuous heparin flush were used to ensure the patency of the direct bypass branch for anastomosis and the indirect branch for EDAS during the operation. Therefore, the separation of the STA branch should also include the protection of small skin branches for subsequent irrigation with the heparin needle. In addition, the thicker adventitia or fascia adhesion layer of the STA was removed as far as possible, which was beneficial to straighten possible vascular turns, use papaverine when vasospasm occurred, and improve blood perfusion through the graft artery.

After cerebral revascularization for patients with MMD, the scalp blood supply was affected, which easily caused skin infection or necrosis [31]. According to previous literature, the incidence of incision disorders relied on the revascularization strategy and skin incision shape, with the percentage of 14.3% for STA-MCA/EDAS which demanded surgical revision, and a complete Y incision giving the worst results, harboring the approximate percentage of 4.2% [32]. In our research, the incidence of scalp infection was approximately 3.3%, including 1.2% in the STA-MCA/EDAS group and 6.0% in the EDAS group. Previous reports reported that diabetes was a potential risk factor for scalp infection [33]. It was important to strengthen blood glucose monitoring in diabetic patients before surgery, reduce unnecessary burning and coagulation of scalp blood vessels during surgery, cover appropriate artificial dura mater, and suture the temporal muscle fascia tightly after surgery, which was able to reduce postoperative cerebrospinal fluid leakage and the incidence of scalp infection or necrosis. Besides, the high tension of the incision suture should also be avoided. Scalp infection and necrosis after revascularization were difficult to treat with the anxiety of damaging the underlying STA, which could also cause intracranial infection or operation failure if not handled properly. For special MMD patients with skin healed poorly, extensive care of the incision should be emphasized. For patients with subcutaneous effusion, it should be actively treated, and skin flap transplantation could also be performed if necessary.

It had been reported in published reports that the incidence of epilepsy after cerebral revascularization in patients with MMD was about 18.9%, which was mainly related to the increased cerebral perfusion or potential CHS for direct or combined bypass, which caused the erethism increased of cerebral cortex, and possible temporal muscle compression for patients with pure EMS or EDMS [34, 35]. However, other studies also reported that epilepsy in MMD patients was only an external manifestation of cerebral ischemia or hemorrhagic events, which was different from those with typical seizures [35, 36]. In addition, previous literature believed that the occurrence of postoperative epilepsy for patients with MMD was closely related to the preoperative epilepsy, which was an independent risk factor for epilepsy recurrence [37]. In our study, no patients were observed to have seizures, which was different from previous literature. We speculated that the low incidence of epilepsy was a feature of cerebral revascularization based on STA, which was associated with careful evaluation of hemispheric cerebral hemodynamics before surgery to design individualized surgical plans, intraoperative treatment and fixation of the superficial temporal artery fascia layer to reduce the compression of the bone flap on the blood vessel, routine use of anti-epileptic drugs (sodium valproate or levetiracetam), postoperative extensive blood pressure control and proper rehydration management, etc. [24]. In addition, only 2 MMD patients were observed with epilepsy before surgery, which could contribute to less recurrence of postoperative seizure.

According to a published report, the annual risk of stroke for the cerebral hemisphere with conservative treatment was 3.2% ~ 19.6% per year, and the 5-year cumulative rebleeding risk for MMD patients with hemorrhage was approximately 16.9% [27, 38]. Other studies from North America and Europe had also shown that the 5-year cumulative stroke incidence for MMD patients with hemodynamic damage was from 40 to 80% [39–41]. Feghali et al. reported that race phenotypic distinctions were related to the natural course of MMD, with a comparatively more progressive course for Asians [42]. Cerebral revascularization was an effective way to improve hemodynamic perfusion and prevent the recurrence of ischemic events, but there was still a controversy regarding the prevention of hemorrhagic events [8, 43]. In this study, the risk of newly developed stroke events after revascularization in the combined group was 15.41% (3.69% per person year), including 11.3% in ischemia and 4.3% in rebleeding. However, the incidence of new stroke attacks in the EDAS alone group was approximately 16.67% (3.92% per person year), including 12.7% in ischemia and 2.8% in rebleeding. Thus, this novel STAPC bypass could prevent the new onset of stroke and reduce the risk of mid-term and long-term stroke for patients with MMD.

Our research had several limitations listed as follows. First, the treatment of STA was the core of revascularization for MMD in our center. When stripping the adventitia and outer layer tissue of STA, pulling and tearing the STA should be minimized, which could reduce its damage. Second, our research only included MMD patients undergoing unilateral surgery, whereas patients with bilateral surgery or with previous surgical history were excluded, which could not truly reflect the overall surgical prognosis of MMD. Third, our study only included 303 patients, of whom some were lost during follow-up, and clinical follow-up was incomplete. Fourth, this was not randomized research. Patients in the STA–MCA/EDAS group and the EDAS-alone group were not randomly assigned, which inherently reflect different hemodynamic conditions. Fifth, pre-infarction stage based on CTP was a semi-quantitative measurement of the hypoperfusion status with no specific value given as a criterion [10]. Whether clinical symptoms align with cerebral perfusion status deserves further research. Finally, this was an observational study. Future multicenter prospective studies are warranted to thoroughly validate the clinical applicability and impact of STAPC for MMD.

## Conclusions

Cerebral revascularization using STAPC was an acceptable surgical strategy, acting as an important role in preventing stroke recurrence for the treatment of MMD patients. Although the role of STAPC in STA-MCA/EDAS and EDAS bypass remained incompletely understood, its significance in sustaining long-term stroke recurrence deserved further research.

## Supplementary Information

Supplementary Material 1.

## Data Availability

The datasets generated during and/or analyzed during the current study are available from the first author on reasonable request (Zhiyong Shi, szy1195156829@aliyun.com).

## Abbreviations

STAPC: Superficial temporal artery patency concept
MMD: Moyamoya disease
EDAS: Encephalo-duro-arterio-synangiosis
CTP: CT perfusion
CT: Computed tomography
CTA: Computed tomography angiography
EC-IC: Extracranial-intracranial
STA-MCA: Superficial temporal artery-middle cerebral artery
MDU: Microvascular doppler ultrasonography
ICG: Indocyanine Green
DSA: Digital subtraction angiography
MMS: Moyamoya syndrome
rCBV: Regional cerebral blood volume
rCBF: Regional cerebral blood flow
MTT: Mean transit time
TTP: Time to peak
