# Supplementary material for: A novel superficial temporal artery patency concept of cerebral revascularization for patients with moyamoya disease: a multicenter study
Source: Chin Neurosurg J. 2026 Feb 26;12:5. doi: 10.1186/s41016-025-00424-4 (PMC12937568; doi:10.1186/s41016-025-00424-4)
Supplement: Supplementary file 1 — Supplementary Material 1. [file 41016_2025_424_MOESM1_ESM.zip › Supplementary material.docx]

**Tips and illustrated cases**

**1. Incision shape.** The incision of the skin relied on the combination of natural course of STA and the range of hairline. STA bifurcation and hairline were both important landmarks for the separation range and protection of MMA. In principle, the incision should run along the STA, but should not exceed the hairline. When a single branch of STA was chosen, the larger branch of STA was harvested, with a linear incision. When two branches were demanded, the incision was made along the course of STA, with the classic Y-shaped incision mostly used. However, for patients with STA or its branches out of hairline, special incision was used, such as inverted hook shape. Whatever the incision was chosen, STA should be separated subcutaneously to zygomatic arch to increase its flexibility and available length for bypass (**Supplementary Figure 1**). In addition, protection of small skin branches for subsequent irrigation with noninvasive needle should also be included during the separation of STA and unnecessary injury should be avoided.

**2. Protection of meningeal compensation.** For MMD patients with compensatory perfusion from MMA, intraoperative protection was important to reduce the incidence of postoperative infarction caused by iatrogenic injury during craniotomy (**Supplementary Figure 2**). For patients with frontal compensation from anterior branch of MMA, the craniotomy should be relatively backward. For patients with compensation from submeningeal branch of MMA, the craniotomy should be more anterosuperior. For cases with anterior and posterior branches of MMA, craniotomy was often located between two branches of arteries. It was important to locate the STA bifurcation and its distance to compensatory MMA based on preoperative DSA, and auxiliary ruler was available when necessary (**Supplementary Figure 3**).

**3. Treatment of STA.** The STAPC was to deal with STA and keep its patency. Dissection of adventia stripping treatment was to achieve the STA as "naked artery", achieving a status of STA with a relatively less adventitia, which was able to elongate, straighten the STA, less stimulation triggered by soft tissue compression, and increase the blood supply for brain^13^. However, any operation of STA could contribute to the thrombus formation and vasospasm. The common purpose of “naked artery” flushed through its small branches with non-invasive heparin needle, was to keep STA unobstructed. Different types of non-invasive needle flushing and expanding STA through its small stump was able to reduce the possibility of thrombus formation. When the adventitia of “naked artery” was stripped as far as possible, patency of STA was confirmed by blood return with heparin needles flushing and intraoperative indocyanine green fluorescence angiography (ICG) application (**Supplementary Figure 4**). Afterwards, STA was for anastomosis with MCA or placed on the surface of brain with its natural course as well as possible.

**Figure legends**

**Supplementary Figure 1.** A summary of skin incision shape based on the combination of natural course of STA on preoperative DSA, palpation of STA and the height of hairline. Classic long Y-shaped incision was mostly used (A). For patients with STA or its branches out of hairline, inverted hook shaped incision was more common to use (B). Sometimes, the straighten incision along the parietal branch of STA was also an option (C).

**Supplementary Figure 2.** Illustrated case of postoperative cerebral infarction with intraoperative iatrogenic injury of meningeal branch when operating EDAS. The submeningeal branch played a compensatory role in blood supply for brain based on preoperative DSA (A). The brain surface was covered by STA and patency of bypass was achieved with ICG (B, C and D). Postoperative MRI and CT scans confirmed right occipitotemporal infarction (E, F, G and H).

**Supplementary Figure 3.** Avoidance and protection of meningeal compensation. Evaluation of compensation from MMA or its branches based on preoperative DSA was important to reduce the incidence of postoperative infarction caused by iatrogenic injury. For patients with frontal compensation from anterior branch (A and B), or submeningeal branch (C), or both anterior and posterior branches of MMA (D), the choice of burr hole and the range of craniotomy were different.

**Supplementary Figure 4.** Treatment of STA. Different types of non-invasive needle flushed the branch of STA for combined bypass (from A to D), which was able to reduce the possibility of thrombus formation and vasospasm of STA. For one case with vasospasm of STA (E and F), the branch of STA was flushed with a thick non-invasive needle and vasospasm relieved thereafter (G and H).
