# Supplementary figures and images for: A novel superficial temporal artery patency concept of cerebral revascularization for patients with moyamoya disease: a multicenter study
Source: Chin Neurosurg J. 2026 Feb 26;12:5. doi: 10.1186/s41016-025-00424-4 (PMC12937568; doi:10.1186/s41016-025-00424-4)

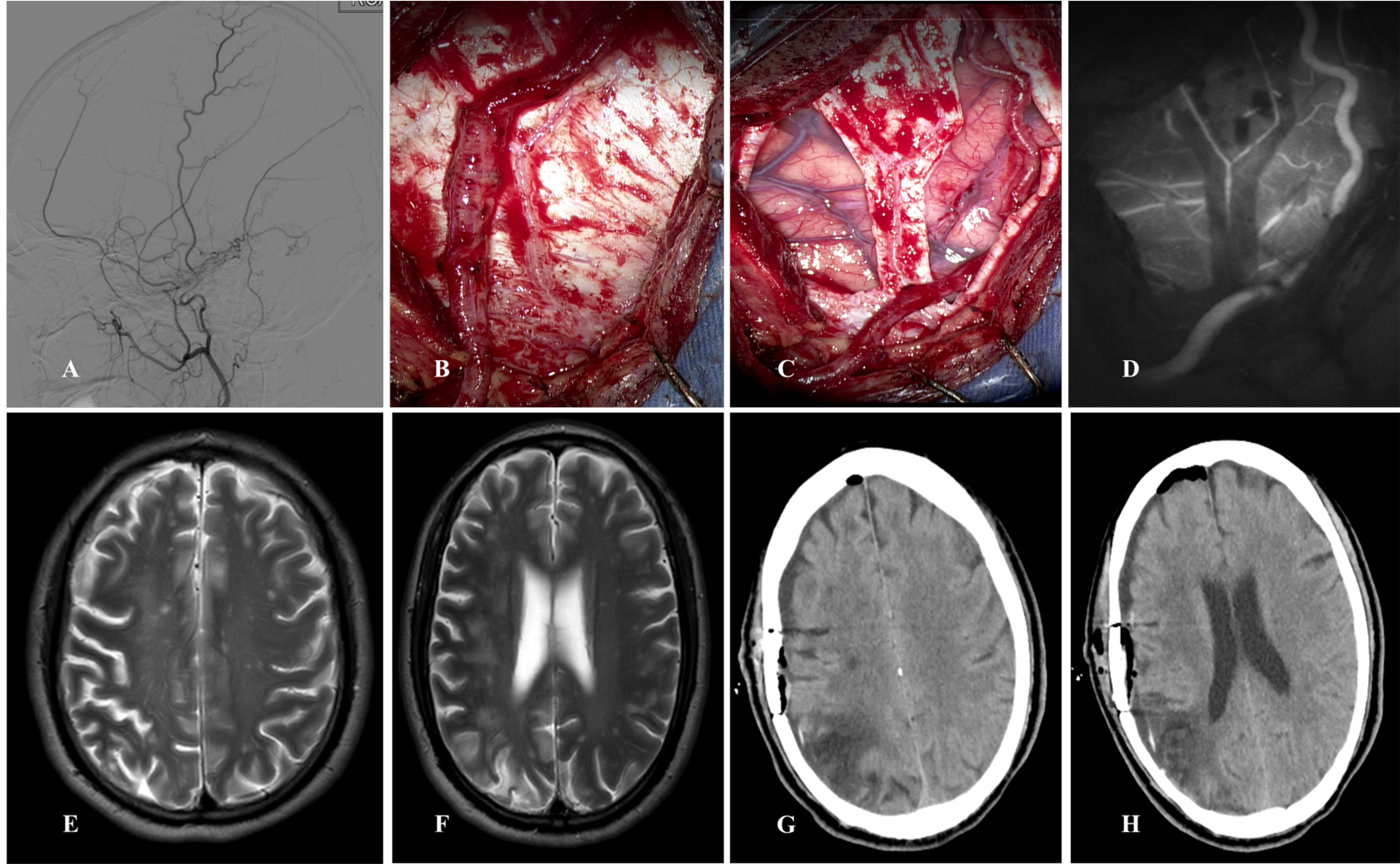

Supplement: Supplementary file 1 — Supplementary Material 1. [file 41016_2025_424_MOESM1_ESM.zip › Supplementary Figure 3.tif]

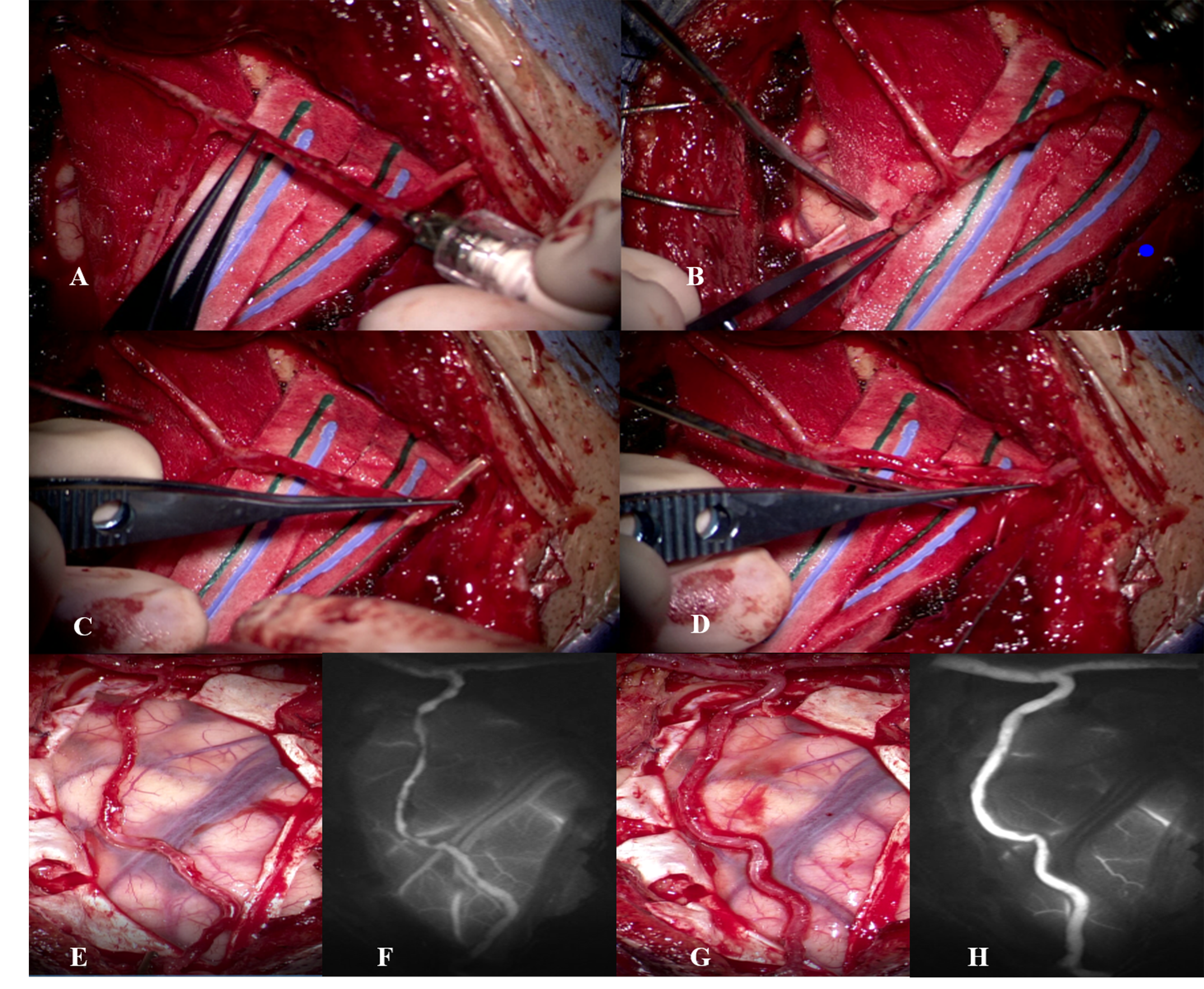

Supplement: Supplementary file 1 — Supplementary Material 1. [file 41016_2025_424_MOESM1_ESM.zip › Supplementary Figure 4.tif]

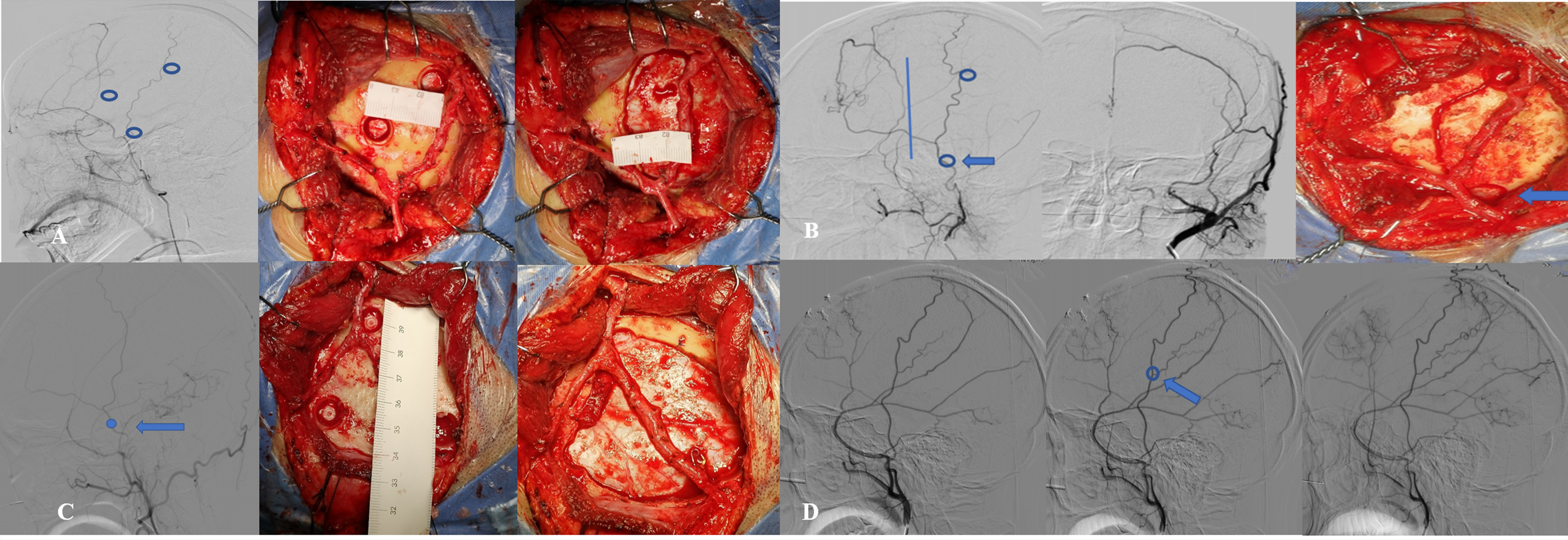

Supplement: Supplementary file 1 — Supplementary Material 1. [file 41016_2025_424_MOESM1_ESM.zip › Supplementary Figure 2.tif]

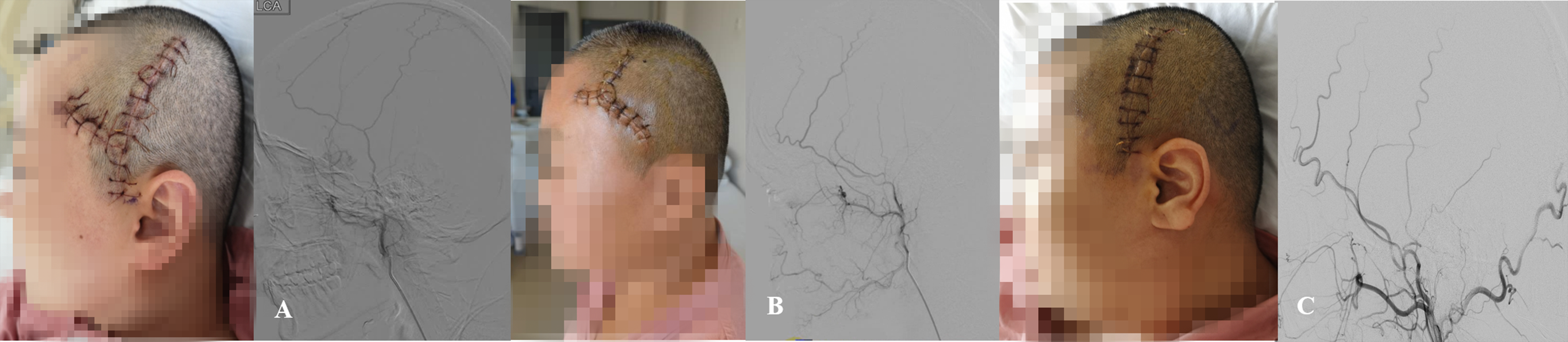

Supplement: Supplementary file 1 — Supplementary Material 1. [file 41016_2025_424_MOESM1_ESM.zip › Supplementary Figure 1.tif]
